# Outcomes of reablement and their measurement: Findings from an evaluation of English reablement services

**DOI:** 10.1111/hsc.12814

**Published:** 2019-08-01

**Authors:** Bryony Beresford, Emese Mayhew, Ana Duarte, Rita Faria, Helen Weatherly, Rachel Mann, Gillian Parker, Fiona Aspinal, Mona Kanaan

**Affiliations:** ^1^ Social Policy Research Unit University of York York UK; ^2^ Centre for Health Economics University of York York UK; ^3^ School of Life & Medical Sciences University College London London UK; ^4^ Department of Health Sciences University of York York UK

**Keywords:** economic evaluation, older people, outcomes, reablement, social care

## Abstract

Reablement – or restorative care – is a central feature of many western governments’ approaches to supporting and enabling older people to stay in their own homes and minimise demand for social care. Existing evidence supports this approach although further research is required to strengthen the certainty of conclusions being drawn. In countries where reablement has been rolled out nationally, an additional research priority – to develop an evidence base on models of delivery – is emerging. This paper reports a prospective cohort study of individuals referred to three English social care reablement services, each representing a different model of service delivery. Outcomes included healthcare‐ and social care–related quality of life, functioning, mental health and resource use (service costs, informal carer time, out‐of‐pocket costs). In contrast with the majority of other studies, self‐report measures were the predominant source of outcomes and resource use data. Furthermore, no previous evaluation has used a global measure of mental health. Outcomes data were collected on entry to the service, discharge and 6 months post discharge. A number of challenges were encountered during the study and insufficient individuals were recruited in two research sites to allow a comparison of service models. Findings from descriptive analyses of outcomes align with previous studies and positive changes were observed across all outcome domains. Improvements observed at discharge were, for most, retained at 6 months follow‐up. Patterns of change in functional ability point to the importance of assessing functioning in terms of basic and extended activities of daily living. Findings from the economic evaluation highlight the importance of collecting data on informal carer time and also demonstrate the viability of collecting resource use data direct from service users. The study demonstrates challenges, and value, of including self‐report outcome and resource use measures in evaluations of reablement.

**What is known about this topic**

- Many western countries’ reablement services are core to strategies to support older people remaining in their homes and limit demand on publicly funded services.
- More robust evaluations of reablement are required to confirm the current view that reablement achieves these objectives.
- Existing evaluations have typically been very limited in the outcomes assessed and, typically, do not include self‐reported outcomes.

**What this paper adds**

- It reports a prospective cohort study which predominantly used self‐reported outcome measures, including outcome domains not previously evaluated.
- It reports a newly developed tool to collect data on resource use.
- Drawing also on findings from previous studies, implications for future evaluations are discussed with respect to measuring outcomes and resource use.

## 1 INTRODUCTION

### 1.1 Background

Over recent years reablement – or restorative care – has increasingly featured within some western governments’ approaches to addressing the care and support needs of older people (Aspinal, Glasby, Rostgaard, Tuntland, & Westendorp, 2016). Delivered in a person's usual place of residence, reablement is a time‐limited, person‐centred intervention. Its aim is to restore self‐care and daily living skills and to support access to, or reconnection with, the local community and social and leisure activities (Tessier, Beaulieu, McGinn, & Latulippe, 2016). Individuals are referred when there is a loss of functioning and independence in managing activities of daily living that, if left unaddressed, will result in increased demands for community‐based services, or necessitate a move to residential care (Cochrane et al., 2016; National Audit of Intermediate Care, 2018; National Institute For Health And Care Excellence, 2017). This may arise following an acute inpatient stay or due to (gradual) loss of abilities, motivation and confidence to engage in and manage everyday activities and tasks. Differences exist – within and between countries – in models of service delivery (e.g. skill mix, organisational setting, operational delivery characteristics; Aspinal et al., 2016; Beresford et al., 2019). In addition, there may be differences in the extent to which provision fully adheres to the concept of reablement and includes reconnecting with social networks (so called “comprehensive reablement”), or is limited to functional reablement Beresford et al. (2019).

In England, reablement comprises an assessment by a specialist practitioner during which person‐centred goals are co‐created with the service user. This is followed by a time‐limited period (typically 4–6 weeks) in which trained workers conduct home visits in order to support the achievement of these goals through the regaining of functional skills and/or identifying new ways of carrying out their activities of daily living. The focus is on “doing with”, in contrast to the traditional, home‐care approach of “doing for” or “doing to” (Metzelthin et al., 2017; Resnick et al., 2016). Frequency and duration of home visits is expected to decrease over the intervention period. Equipment or minor housing adaptations may be sourced to support achievement of outcomes.

Existing evidence indicates reablement results in improved functioning, quality of life and/or reduced demands on services. To date, however, evaluations have not been of sufficient quality for robust conclusions to be drawn regarding effectiveness and cost‐effectiveness and the need for high‐quality trials is acknowledged (Cochrane et al., 2016; National Institute For Health And Care Excellence, 2017). Investment in reablement – at a policy and resource level – adds to the pressing need to improve and extend the existing evidence base.

This paper reports a prospective cohort study of older people receiving reablement in England. It was commissioned by the English government's National Institute for Health Research who issued a call for proposals to investigate different models of service delivery. This was in response to the fact that, in England, reablement services are universal but different delivery models exist (Parker, 2014). As reported in the methods section, the study did not fulfil all its objectives; however, it did generate new and important evidence on a range of outcomes associated with reablement and the use of self‐report measures in this context.

## 2 METHODS

An overview of the method is presented below, a full account is available (Beresford et al., 2019).

### 2.1 Study design

The study design was a prospective cohort study comparing outcomes and resource use for individuals referred to one of three reablement services, each representing a different model of service delivery (e.g. inclusion of OT within team, reablement only caseload versus mixed caseload (i.e. reablement and home care)). Descriptions of service models are available (Beresford et al., 2019). Data were collected at entry to the service (T_0_), discharge (T_1_) and 6 months post discharge (T_2_).

Significant under‐recruitment in two research sites (*n* = 14 and 29, respectively, compared to 139 in third site) due to service throughput being much slower than anticipated, and no option to extend the study or add new research sites, meant a comparison of service models was not possible. (For a detailed account, see Beresford et al., 2019). However, a descriptive analysis of combined outcomes and resource use data was conducted.

Ethical approval was received from a National Health Service (NHS) Health Research Authority Research Ethics Committee (Reference: 15/NE/0299).

### 2.2 Setting

The study recruited from three statutorily funded adult social care reablement services located in different regions in England. Recruitment took place between October 2016 and May 2017.

### 2.3 Participants

Study inclusion criteria were that participants had been accepted into one of the reablement services acting as a research site. Individuals lacking the capacity to give informed consent (as judged by reablement service assessors or research team) were excluded.

### 2.4 Recruitment

At the reablement service's assessment visit (taking place within 3 days of referral), the assessor briefly introduced the study and sought consent for the research team to make contact. Those consenting to contact received a telephone call from the research team (i.e. the “local” researcher based in research site). If agreed, a home visit was arranged to further discuss participation and, if willing, take consent and collect T_0_ data. A £10 shopping voucher (multi‐store, high street/online) supported recruitment and retention.

### 2.5 Data collection

Self‐reported outcomes data were collected via home visits. Participants chose whether to self‐complete, or have measures provided verbally and responses recorded by the researcher. Some T_2_ data were collected via post. Assessors within the reablement services completed the Barthel Index.

### 2.6 Outcomes

Selection of outcome measures was informed by: (a) a desire to include self‐reported outcomes, (b) the lack of research infrastructure within reablement services allowing only minimal data collection by practitioners; (c) a previous evaluation of English reablement services (Glendinning et al., 2010).

#### 2.6.1 EQ‐5D‐5L

A standardised self‐report measure assessing health‐related quality of life (HRQoL) on the dimensions of mobility, self‐care, usual activities, pain/discomfort and anxiety/depression and according to five levels of severity (no problems, slight moderate, severe and extreme problems; Brooks, 1996; Herdman et al., 2011; The EuroQol Group, 1990). HRQoL profiles were converted into a single index score using the UK tariff (Devlin, Shah, Feng, Mulhern, & Hout, 2018). Index scores range from −0.285 (for extreme problems on all dimensions) to 0.950 (no problems in any dimension). In addition, a visual analogue scale (EQ‐VAS) records self‐rated health on a scale from 0 “worst imaginable health state” to 100 “best imaginable health state”.

#### 2.6.2 Adult Social Care Outcomes Toolkit's SCT‐4

A standardised self‐report measure assessing social care–related quality of life across eight domains: control over daily life; personal cleanliness and comfort; food and drink; personal safety; social participation and involvement; occupation; accommodation cleanliness and comfort; and dignity (Malley et al., 2012). For each domain, respondents select one of four options: ideal state, no needs, some needs and high needs. The total score is converted into an index score using preference‐based weights valued using best–worst scaling and time trade off in an adult general population sample.

#### 2.6.3 General Health Questionnaire

A self‐report measure in which respondents rate current mental health compared to their usual state. Items cover inability to carry out normal functions and the appearance of new and distressing emotional states (Goldberg, 1972). For each item, respondents choose one of four response options: better than usual, same as usual, less than usual and much less than usual. The standard method of scoring was used with positive answers (better/same as usual) scored as 0 and negative answers (less/much less than usual) scored as 1. The maximum total score is 12, with a higher score indicating more severe mental health difficulties.

#### 2.6.4 Barthel activities of daily living index

A practitioner‐completed 10‐item measure of functional status covering 10 domains of daily living: feeding, bathing, continence (bladder, bowels), transfers (bed/chair, to and from toilet), mobility (level surface, stairs) and personal grooming (Mahoney & Barthel, 1965). Each domain is rated on a scale from no functioning to independent functioning. The number of points on the scale varies between items and ranges between 2 and 4 points. Scores assigned to each point on the scale increase by 5‐point intervals (e.g. 0–5–10–15). Total scores can range from 0 (no functioning) to 100 (independent functioning).

#### 2.6.5 Nottingham Extended Activities of Daily Living Scale

A self‐report measure of functional ability with respect to mobility, kitchen tasks, domestic tasks and leisure. Comprising 22 items, it captures a wider assessment of functioning than the Barthel Index (Nouri & Lincoln, 1987). Respondents evaluate the extent to which they can accomplish each functional task scoring 0 (not able/with help) or 1 (on their own/on their own with difficulty). A total score is calculated ranging between 0 (no independence) and 22 (maximum independence).

### 2.7 Resource use

A self‐report questionnaire (Services and Care Pathway Questionnaire [SCPQ]) developed for the study collected data on: use of hospital, community healthcare, social care and voluntary services, informal (unpaid) care and private out‐of‐pocket costs. Total costs were calculated by multiplying the number of times each resource was used by its unit cost for the financial year 2016. Further information on the development of the SCPQ and how costs were calculated are available (Beresford et al., 2019). Since the period of recall was different at each follow‐up point, resource use and the costs were rescaled to mean use per week.

### 2.8 Statistical analysis

STATA 14.2 was used (StataCorp, 2015). Descriptive statistics for socio‐demographic characteristics, outcome measures and resource use and costs at T_0_, T_1_ and T_2_ were generated. Means and standard deviations (*SD*) were reported for continuous variables and counts and percentages for categorical variables. The characteristics of individuals retained to the study at T_1_ and T_2_ were compared to those lost to follow‐up using *t* test for continuous variables and Pearson's Chi‐square test for categorical variables. We also tested for differences in outcomes at T_0_, T_1_ and T_2_ according to the reason for referral to reablement (remain at home vs. return home (i.e. discharged home from hospital)).

A descriptive analysis of outcomes generated mean and standard deviation statistics for total scores for T_0_, T_1_ and T_2_ samples. A domain‐level descriptive analysis of quality‐of‐life outcomes was also conducted. For EQ‐5D‐5L, response options were collapsed into three categories of perceived severity of problems: severe/extreme, moderate or no/slight. For Adult Social Care Outcomes Toolkit (ASCOT) SCT‐4, response options were collapsed into two categories of perceived need: needs met (ideal state or no needs reported) or unmet needs (some needs or high needs).

The next stage was a descriptive analysis of changes in outcome for those where data were available for the following pairs of time points: T_0_ to T_1_, T_0_ to T_2_, T_1_ to T_2_. First, mean and standard deviation statistics were generated for total scores and tests of statistical significance and effect size calculated. Second, we explored direction of change in outcomes at an individual level. Study participants were allocated to one of three categories: improved, no change, deteriorated. Frequency counts were used to describe the distribution of the sample according to these categories.

We also explored the impact of mode of data collection on response rate for outcomes collected at T_2_ (where some study questionnaires were delivered postally rather than via a home visit).

We considered a *p*‐value of 0.05 to be statistically significant and provided 95% confidence intervals (CI) for the estimates.

## 3 RESULTS

### 3.1 Recruitment, retention and impact of mode of data collection

**Figure 1:**
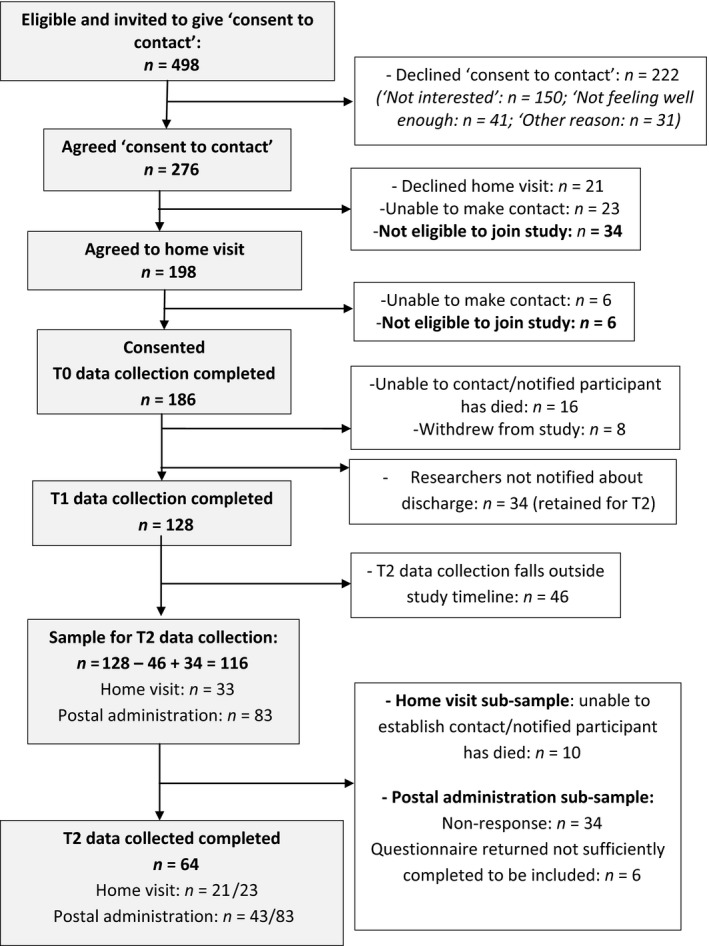
Flow chart of recruitment and retention

Recruitment and retention is set out in Figure 1. One hundred and eighty‐six individuals were recruited, representing just over 40% of those approached (*n* = 186/458). Predominant reasons for refusing consent to contact chosen from a pre‐determined list were “not interested” (67.6%) and “not feeling well enough” (18.7%). T_1_ data collection was not achieved for 34 participants due to research sites failing to notify the research team about a discharge. Taking this into account, T_1_ retention where data collection was attempted was 84% (128/152). Loss to the study at T_1_ was principally due to a participant having died or the researcher being unable to re‐establish contact. This may have been due to death, readmission to hospital or move to residential care which research sites were unaware of, or did not report to the research team. Eight participants chose to withdraw at this stage.

At T_2_, 46 study participants were not followed up because T_2_ occurred after the study closed. Loss of local research staff associated with closure of the study meant postal administration of questionnaires was used for some study participants. The response rate among those where T_2_ data collection was attempted via a home visit was 91% (*n* = 21/23). Postal administration yielded a response rate of 59% (*n* = 59/83); however, six questionnaires had only been completed very partially and could not be included in analyses.

### 3.2 Sample characteristics

Characteristics of the recruited sample (T_0_) and T_1_ and T_2_ samples are set out in Table 1. No statistically significant differences in these characteristics were observed between T_0_, T_1_ and T_2_ samples.

**Table 1:** Characteristics of T_0_, T_1_ and T_2_ sample

	T_0_ *N* (%)	T_1_ *N* (%)	T_2_ *N* (%)
Total	186	128	64
Gender			
Female	119 (64)	87 (68)	44 (69)
Male	67 (36)	41 (32)	20 (31)
Lives alone			
No	79 (42)	51 (40)	27 (42)
Yes	107 (58)	77 (60)	37 (58)
Reason for referral			
Return home	75 (40)	53 (41)	22 (34)
Remain at home	111 (60)	75 (59)	42 (66)
Informal carer involved			
No	20 (11)	15 (12)	7 (11)
Yes	164 (89)	113 (88)	57 (89)
Number of comorbidities			
None	67 (36)	46 (36)	28 (44)
1	79 (42)	55 (43)	25 (39)
2 or more	40 (22)	27 (21)	11 (17)
Age (years)			
Mean (*SD*)	80.85 (9.1)	80.83 (9.0)	81 (8.8)
Median	82	82	83
Range: min, max	51, 102	51, 102	51, 98

### 3.3 Duration and intensity of reablement

The planned duration of reablement was typically 6 weeks (*n* = 170; 91%) and involved 12 sessions on average per week (*SD* = 7). In England, six weeks is, formally, the maximum duration for which service users do not have to pay for the service. Actual duration was similar across research sites and was, on average, 3.9 weeks.

### 3.4 Outcomes

There were no statistically significant differences at baseline (T_0_) in mean outcome scores for the recruited sample and those retained at T_1_, nor between those referred for support to return home from hospital versus where the referral was to support remaining at home. Those retained at T_2_ had significantly higher (better) scores on the Barthel Index, Nottingham Extended Activities of Daily Living Scale (NEADL) scale and General Health Questionnaire (GHQ‐12) at T_0_ than the total sample recruited.

#### 3.4.1 Descriptive statistics: total scores

Table 2 displays descriptive statistics for scores on outcome measures observed at T_0_, T_1_ and T_2_. Differences in mean score between T_0_ and T_1_ are all in a positive direction. For EQ‐5D‐5L, EQ‐VAS and GHQ‐12, the difference between T_1_ and T_2_ mean scores is smaller than between T_0_ and T_1_ but remains in the same direction. For the ASCOT‐SCT4 the T_2_ mean score was slightly lower than the T_1_ mean score. For the NEADL scale, the size of the difference in mean score was greater between T_1_ and T_2_ than T_0_ and T_1_. Mean scores at T_1_ and T_2_ for Remain at Home and Return Home sub‐samples were not significantly different.

**Table 2:** Differences in outcome scores observed T_0_, T_1_ and T_2_

	T_0_	T_1_	T_2_
EQ‐5D‐5L (2017 tariff)			
Sample size	(*n* = 186)	(*n* = 128)	(*n* = 61)
Mean (*SD*)	0.51 (0.23)	0.67 (0.24)	0.69 (0.26)
EQ‐VAS			
Sample size	(*n* = 185)	(*n* = 128)	(*n* = 61)
Mean (*SD*)	51.83 (20.23)	63.52 (20.46)	68.77 (20.55)
ASCOT SCT‐4			
Sample size	(*n* = 184)	(*n* = 128)	(*n* = 59)
Mean (*SD*)	0.71 (0.17)	0.82 (0.15)	0.80 (0.17)
Barthel Index			
Sample size	(*n* = 130)	(*n* = 133)	
Mean (*SD*)	71.69 (17.02)	80.45 (20.28)	
NEADL scale			
Sample size	(*n* = 184)	(*n* = 128)	(*n* = 64)
Mean (*SD*)	9.65 (5.48)	10.40 (4.46)	13.22 (6.27)
GHQ‐12			
Sample size	(*n* = 185)	(*n* = 128)	(*n* = 62)
Mean (*SD*)	4.14 (2.85)	2.42 (2.60)	2.10 (2.65)

#### 3.4.2 Descriptive statistics: EQ‐5D 5L and ASCOT SCT‐4 domains

##### EQ‐5D‐5L

**Figure 2:**
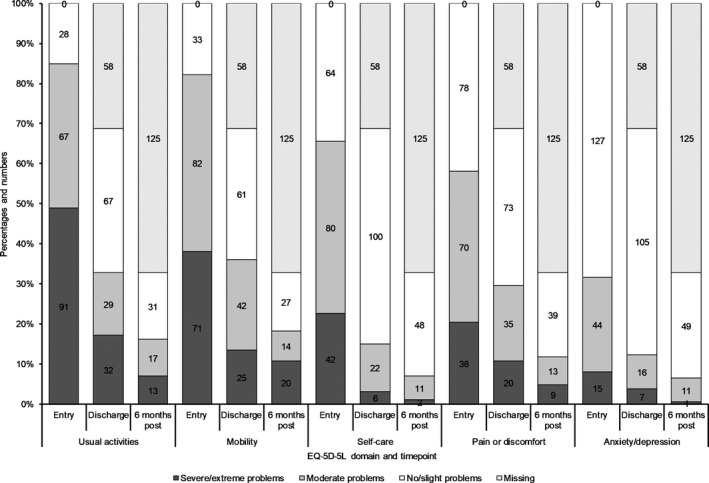
EQ‐5D‐5L domains: distribution of sample in terms of perceived severity of problem: entry into service, discharge and 6 months post discharge

At T_0_, over 80% of the sample reported severe or moderate problems with achieving usual activities and being mobile, see Figure 2. Around two‐thirds reported severe or moderate problems with self‐care, with a slightly smaller proportion reporting problems with pain/discomfort. The domain where the fewest respondents reported problems was anxiety/depression.

At T_1_, around half of the sample reported no/slight problems with usual activities and mobility, and more than three quarters reported no/slight problems with self‐care. These proportions remained around the same at T_2_. The proportions of respondents reporting severe or moderate difficulties with pain/discomfort and anxiety/depression are relatively stable across these time points.

##### ASCOT‐SCT4

**Figure 3:**
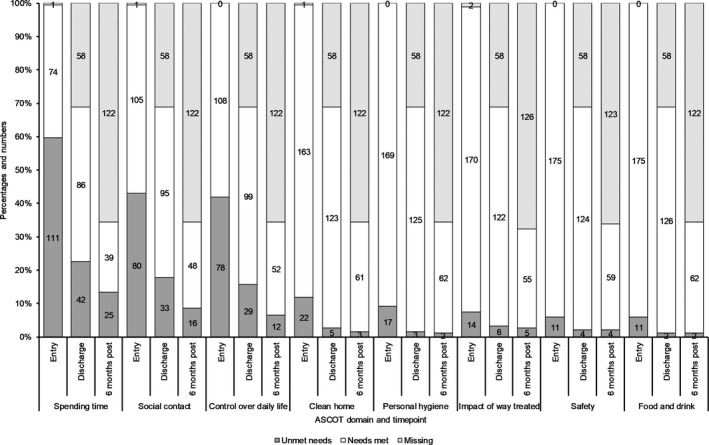
Adult Social Care Outcomes Toolkit (ASCOT) SCT4 domains: proportions reporting needs met versus unmet needs at entry, discharge and 6 months post discharge

At T_0_, domains where unmet needs most likely to be reported were the way people spent their time, level of social contact and feeling in control over daily life, see Figure 3. At T_1_, the proportion reporting unmet needs in these domains was smaller. This was also observed at T_2_ for social contact and control over daily life. For the remainder of the domains, at any time point only a small minority of the sample reported unmet need.

#### 3.4.3 Changes in outcomes

Table 3 presents changes in outcomes for study participants where data are available for the following pairs of time points: T_0_ and T_1_, T_0_ and T_2_ , and T_1_ and T_2_.

**Table 3:** Change in outcomes^a^: T_0_ to T_1_, T_0_ to T_2_ and T_1_ to T_2_

	T_0_–T_1_	T_0_–T_2_	T_1_–T_2_
EQ‐5D‐5L (2017 tariff)	(*n* = 128)	(*n* = 61)	(*n* = 49)
Mean score	T_0_ = 0.51; T_1_ = 0.67	T_0_ = 0.54; T_2_ = 0.69	T_1_ = 0.67; T_2_ = 0.69
Difference in mean score	0.15	0.15	−0.02
95% CI	0.12, 0.18	0.097, 0.20	−0.086, 0.03
*p* value	<.001	<.001	.451
Effect size^b^	0.831	0.728	−0.108
EQ‐5D (VAS)	(*n* = 127)	(*n* = 61)	(*n* = 51)
Mean score	T_0_ = 51.58; T_1_ = 63.39	T_0_ = 51.00; T_2_ = 68.77	T_1_ = 65.02; T_2_ = 68.24
Difference in mean score	11.81	17.77	3.22
95% CI	8.10, 15.52	11.94, 23.60	−3.49, 9.92
*p* value	<.001	<.001	.340
Effect size^b^	0.559	0.780	0.135
ASCOT SCT‐4	(*n* = 128)	(*n* = 59)	(*n* = 47)
Mean score	T_0_ = 0.73; T_1_ = 0.82	T_0_ = 0.70; T_2_ = 0.80	T_1_ = 0.791; T_2_ = 0.792
Difference in mean score	0.09	0.10	0.002
95% CI	0.06, 0.11	0.05, 0.15	−0.04, 0.04
*p* value	<.001	<.001	.928
Effect size^b^	0.641	0.540	0.013
Barthel Index	(*n* = 96)	Barthel Index not collected at T_2_.	
Mean score	T_0_ = 72.4; T_1_ = 80.1		
Difference in mean score	7.71		
95% CI	4.03, 11.39		
*p* value	.001		
Effect size^b^	0.424		
NEADL Scale	(*n* = 128)	(*n* = 64)	(*n* = 52)
Mean score	T_0_ = 9.67; T_1_ = 10.40	T_0_ = 11.58; T_2_ = 13.22	T_1_ = 11.50; T_2_ = 13.29
Difference in mean score	0.73	1.64	1.79
95% CI	−0.06, 1.51	0.17, 3.11	0.55, 3.03
*p* value	.071	.029	.006
Effect size^b^	0.161	0.279	0.401
GHQ‐12	(*n* = 128)	(*n* = 62)	(*n* = 50)
Mean score	T_0_ = 3.95; T_1_ = 2.42	T_0_ = 3.89; T_2_ = 2.10	T_1_ = 2.62; T_2_ = 2.06
Difference in mean score	−1.53	−1.79	−0.56
95% CI	−1.96, −1.11	−2.46, −1.11	−1.28, 0.16
*p* value	<.001	<.001	.123
Effect size^b^	−0.629	−0.67	0.222

Note

Difference in mean scores between time points are presented with corresponding: *p*‐values, 95% CI and effect size. Mean scores at each time point are also presented.

a For all measures except GHQ‐12, higher scores = better outcomes. For GHQ‐12, it is the reverse.

b Cohen's *d* = (mean_2_ − mean_1_)/standard deviation, (*d* = 0.2 small, *d* = 0.5 medium, *d* = 0.8 large).

Compared to T_0_, at T_1_ a statistically significant improvement in mean score was observed for all outcome measures except the NEADL scale. Comparing T_0_ and T_2_, a statistically significant difference in mean scores was observed for all outcome measures.

Looking specifically at any changes in outcomes after discharge from reablement, a significant difference in mean score at T_2_ compared to T_1_ was observed for the NEADL Scale only. Here, the size of the difference in mean score between T_1_ and T_2_ was larger than that observed between T_0_ and T_1_ (1.79 vs. 1.64).

### 3.5 Direction of change

Table 4 presents the direction of change in scores in terms of the proportions of participants whose scores improved, remained the same or deteriorated.

**Table 4:** Direction of change in scores on outcome measures

Nature of change	T_0_ to T_1_		T_0_ to T_2_		T_1_ to T_2_	
	*n*	%	*n*	%	*n*	%
EQ‐5D‐5L (T_0_–T_1_: *n* = 128; T_0_–T_2_: *n* = 61; T_1_–T_2_: *n* = 49)						
Deterioration	16	12.5	11	18.0	21	42.9
Maintenance	4	3.1	0	0	3	6.1
Improvement	108	84.4	50	82.0	25	51.0
ASCOT SCT‐4 (T_0_–T_1_: *n* = 128; T_0_–T_2_: *n* = 59; T_1_–T_2_: *n* = 49)						
Deterioration	31	24.2	17	*28.8*	21	44.7
Maintenance	4	3.1	0	*0*	3	6.4
Improvement	93	72.7	42	71.2	23	48.9
Barthel Index (T_0_–T_1_: *n* = 63) (not collected at T_2_)						
Deterioration	22	22.9	—	—	—	—
Maintenance	11	11.5	—	—	—	—
Improvement	63	65.5	—	—	—	—
NEADL scale (T_0_–T_1_: *n* = 128; T_0_–T_2_: *n* = 64; T_1_–T_2_: *n* = 50)						
Deterioration	39	30.5	21	32.8	14	26.9
Maintenance	18	14.1	8	12.5	4	7.7
Improvement	71	55.5	35	54.7	34	65.4
GHQ‐12 (T_0_–T_1_: *n* = 128; T_0_–T_2_: *n* = 62; T_1_–T_2_: *n* = 50)						
Deterioration	23	18.0	10	16.1	12	24.0
Maintenance	16	12.5	10		13	26.0
Improvement	89	69.5	42	67.7	25	50.0

At T_1_, an improvement in EQ‐5D‐5L (84.4%), ASCOT SCT‐4 (72.7%), Barthel Index (65.5%) and GHQ‐12 (69.5%) scores compared to T_0_ was observed in a large majority of the sample. The proportion of the sample where NEADL scale scores had improved was smaller (55.5%), but remained at over half of the sample. Across all outcome measures, a deterioration as opposed to no change was more likely to be observed between T_0_ and T_1_. Deterioration was least likely to be observed with respect to EQ‐5D‐5L scores (12.5%), and most likely to be observed for on the NEADL scale (30.5%).

Between T_0_ and T_2_, the majority of participants’ EQ‐5D‐5L and ASCOT‐SCT4 scores had improved (82% and 71.2%); with the remainder deteriorating. In terms of the NEADL scale, over half had improved scores (54.7%) and just under a third's scores had declined (32.8%). Finally, improved scores on the GHQ‐12 were observed for over two‐thirds of the sample (67.7%); of the remainder, equal proportions (16.1%) were observed to have deteriorated or scores were the same as at entry into reablement (T_0_).

In terms of direction of change in outcomes between T_1_ and T_2_, improvements in around half of study participants’ scores on the EQ‐5D‐5L (51%), ASCOT SCT‐4 (48.9%) and GHQ‐12 (50%) were observed at T_2_. With respect to self‐reported functioning (NEADL), improved scores were observed for two‐thirds (65.4%) of study participants at T_2_. A deterioration at T_2_ was less likely to be observed on the GHQ‐12 (24%) than EQ‐5D‐5L (42.9%) and ASCOT SCT‐4 (44.7%).

### 3.6 Resource use and costs

At T_0_, all but one participant completed the SPCQ (*n* = 185). At T_1_ and T_2_, all those remaining in the study completed it. The response rate for all questions was above 90%. Participants generally preferred to have the SCPQ administered as a structured interview rather than self‐complete.

#### 3.6.1 Resource use

Resource use was more frequent before reablement, particularly overnight hospitalisations and care services, see Table 5. Some participants had home adaptations, generally minor. Equipment acquisition was more common, typically before and during reablement. Voluntary service use was very rare throughout the study. Informal care provision was frequent but reduced over time.

**Table 5:** Resource use, standardised to mean use per week

Resource	T_0_			T_1_			T_2_		
	*N*	Mean	*SD*	*N*	Mean	*SD*	*N*	Mean	*SD*
Hospital length of stay, number of nights	158	2.32	2.34	124	0.04	0.27	50	0.16	0.42
Hospital visit without overnight stay, number of visits	174	0.31	0.21	127	0.24	0.34	65	0.18	0.21
Community health care, number of visits	180	2.08	2.35	128	1.19	1.61	62	0.90	1.36
Care services, number of hours	182	3.09	2.51	127	2.10	2.71	65	0.50	1.65
Other social care services, number of times service was used	180	0.92	1.29	123	1.00	1.63	61	0.72	2.77
Voluntary or charity service, number of times service was used	183	0.04	0.16	127	0.02	0.12	64	0.07	0.22
Major home adaptations, number of adaptations	185	0.01	0.03	128	0.01	0.05	66	0.00	0.01
Minor home adaptations, number of adaptations	185	0.04	0.09	128	0.09	0.32	66	0.02	0.04
Equipment, number of equipment items	185	0.24	0.23	128	0.21	0.30	66	0.06	0.09
Informal care, hr	177	23.77	35.76	123	20.03	37.23	56	11.21	27.68

#### 3.6.2 Costs

Costs of healthcare and social care falling on the public sector were greatest prior to reablement, with a large reduction observed in the cost of hospital overnight stays (Table 6). Out‐of‐pocket costs were generally very small throughout the study. Informal care time was a major cost, particularly prior to and during reablement.

**Table 6:** Costs, standardised to mean cost per week

Sector	Cost	At entry to the service			At discharge from the service			At 6 months follow‐up		
		*N*	Mean	*SD*	*N*	Mean	*SD*	*N*	Mean	*SD*
Public^a^	Hospital overnight stays	158	£719	£722	124	£11	£81	50	£52	£138
	Hospital visits	174	£31	£31	127	£29	£46	65	£26	£33
	Community healthcare	180	£27	£28	180	£21	£22	62	£16	£22
	Social care	179	£44	£33	126	£32	£36	61	£10	£27
Out‐of‐pocket^b^	Major home adaptations	184	£0	£1	128	£0	£0	51	£2	£6
	Minor home adaptations	182	£2	£5	127	£3	£8	59	£2	£9
	Equipment	184	£0	£1	127	£0	£0	65	£0	£0
	Community healthcare	181	£13	£67	127	£0	£0	62	£3	£22
	Social care	180	£0	£1	128	£0	£1	53	£0	£1
	Voluntary sector	172	£1	£5	123	£0	£2	58	£0	£1
Other^c^	Major home adaptations	183	£1	£4	127	£0	£2	£1	£1	£3
	Minor home adaptations	182	£32	£145	127	£24	£268	£228	£9	£43
	Equipment	182	£1	£4	128	£2	£9	£13	£1	£2
	Voluntary sector	180	£23	£45	111	£13	£39	£139	£6	£16
	Informal care	177	£374	£562	123	£315	£585	£176	£176	£435

a Public sector costs include the cost of healthcare and social care services funded by the NHS and local authorities’ social services, using national prices.

b Out‐of‐pocket costs include costs paid for privately by the study participants according to their answers to Services and Care Pathway Questionnaire.

c Other costs are the costs of services, house adaptations and equipment, all costed as if these services and items been provided by the public sector, and informal care time valued using the average wage rate in the UK.

## 4 DISCUSSION

Challenges experienced with study set‐up and recruitment – predominantly due to the lack of research support structures within English social care services and slower than anticipated service throughput – meant the study was closed prior to achieving its desired sample size. Consequently, it was not possible to fulfil one of the main objectives – to evaluate and compare different models of delivering reablement. However, a descriptive analysis of outcomes and resource use was possible.

The study offers a number of further contributions. It used outcome measures and a follow‐up time point not previously (or infrequently) used. In contrast to most studies, constraints in research funding and research capacity within services meant we relied primarily on self‐reported outcomes. We also developed a new self‐report tool to assess resource use. Finally, different modes of data collection were tested.

### 4.1 Findings on reablement outcomes and implications for future research

To our knowledge, this study evaluated the widest range of outcome domains including quality of life, functioning and mental health.

In terms of observed changes in outcomes at discharge (T_0_ to T_1_) and at 6 months follow‐up (T_2_), a number of points are highlighted. First, the size and pattern of change varied between outcomes. For health‐related quality of life (EQ‐5D‐5L, EQ‐5D VAS), a significant change in scores representing a large, or medium‐large effect, was observed at discharge with this improvement maintained at 6 months post discharge. A similar pattern was observed for social care–related quality of life (ASCOT SCT‐4) though the effect size was only medium. We note that no guidance currently exists on what constitutes a minimal important change in index score for these measures with this population (van Leeuwen et al., 2015).

One previous study (Glendinning et al., 2010) used (earlier versions of) these measures, investigating outcomes at 12‐month follow‐up in two cohorts: those in receipt of reablement and those receiving home care. Findings from this and our study align in terms of health‐related quality of life. However the previous study did not find a difference in social care–related quality of life between the cohorts at 12 months follow‐up, nor were changes in scores between baseline and 12‐month follow‐up statistically significant. Two other studies (Lewin, De San Miguel, et al., 2013; Tuntland, Aaslund, Espehaug, Forland, & Kjeken, 2015) – both randomised controlled trials comparing reablement with usual care – used alternative measures of quality of life: the COOP/Wonka and the Assessment of Quality of Life Scale (AQoL). Neither report reablement significantly affecting health‐related quality of life at follow‐up time points compared to usual care. Both studies posit a number of explanations for these findings, including the same workers providing reablement and usual care and other limitations in study design. However, these findings do highlight that wider recovery processes, independent of reablement, may be driving or contributing to observed improvements in quality of life.

Inspection of EQ‐5D‐5L and ASCOT SCT4 domain scores raise some interesting issues. While our findings suggest that all EQ‐5D domains are relevant to evaluating the impact of reablement, this is not so for ASCOT SCT4. Just three of the eight domains (activities/occupation, social participation, sense of control over daily life) were reported as problematic by at least 40% of the sample at entry into reablement. All are highly salient to the objectives of reablement and, apart from the “usual activities” domain, capture outcome domains not assessed by the EQ‐5D‐5L. In terms of the remaining ASCOT domains, just 1 in 10, or fewer, participants reported these problematic at entry into reablement. We also suggest caution when interpreting improvements observed at discharge in the “social participation” domain because these might be attributable, to some degree, to the increased level of social contact experienced through the visits of reablement workers. This can be highly valued by service users (Gethin‐Jones, 2013; Beresford et al., 2019).

The study assessed ability to carry out activities of daily living using practitioner‐ (Barthel Index) and self‐report (NEADL scale) measures. The latter has not previously been used to evaluate reablement. It was only possible to administer the Barthel Index at entry into the service and discharge. At discharge, a significant change in score was observed, representing a small–medium effect. This finding aligns with those of two previous trials in Australia which used a modified version of this instrument. In contrast, the difference in mean score on the NEADL scale between T_0_ and T_1_ was not statistically significant. However, a significant change in mean score was observed between T_1_ and T_2_, representing a small effect over this time period and contributing to a small–medium effect between T_0_ and T_2_.

The difference in findings from these two measures is likely to reflect that the Barthel Index measures functioning with respect to the core activities of daily living, while the NEADL scale measures what is defined as *extended (or instrumental)* activities of daily living. Our pattern of results suggests further and broader gains in functioning may be achieved once individuals are discharged from reablement. The absence of a comparator group means we cannot attribute these improvements to reablement and they may, instead or in part, be due to non‐specific recovery processes observed after, for example, a fracture has healed (Tuntland et al., 2015). However, a study which did use a comparator groups found differences between groups in (practitioner‐reported) abilities to carry out extended activities of daily living (favouring the reablement group) were not observed until some months after discharge (Lewin, De San Miguel, et al., 2013).

These findings support wider arguments that: (a) evaluations of reablement should assess functioning with respect to core *and* extended activities of daily living, and (b) longer term follow‐up should be included in study designs. With regard to the first point, tools which measure both core and extended activities of daily living are now being developed (Chen et al., 2012; LaPlante, 2010). Also relevant here are concerns being expressed about the psychometric properties of some existing measures, and their use with populations for whom they were not originally designed (de Morton, Keating, & Davidson, 2008; Tennant, Geddes, & Chamberlain, 1996). These points should inform future decisions about selection of measures of functioning.

An alternative approach to the use of standardised measures, and adopted by a Norwegian RCT of reablement (Tuntland et al., 2015), are clinical, goal‐setting interviews to identify and monitor functional outcomes prioritised by the service user. This approach aligns well with the ethos and objectives of reablement and is common within the field of rehabilitation (Turner‐Stokes, 2009). However, this is only possible if services have capacity to integrate this into their routine practice or evaluations are sufficiently resourced to incorporate this.

Mental health outcomes, assessed using the GHQ‐12, showed a pattern of change similar to that observed for healthcare‐ and social care–related quality of life. A significant change in score was observed between T_0_ and T_1_, representing a medium–large effect, with this change maintained at T_2_. Just one previous study has evaluated impacts on mental health (Lewin & Vandermeulen, 2010). This non‐randomised trial used a measure of morale (Philadelphia Geriatric Center Morale Scale) and reported significant improvements for this outcome at 3 and 12 months follow‐up.

While the objectives (and primary outcomes) of reablement are to restore and/or retain skills which allow individuals to manage everyday living activities as independently as possible (Aspinal et al., 2016), these findings indicate an important secondary effect of reablement. It may be the case that (re)gains in independence and re‐engagement with everyday life achieved through reablement directly cause gains in mental health through, for example, improved self‐worth and self‐efficacy, and the pleasure and satisfaction derived from engaging in meaningful activities. However, other mechanisms may also be at play both during reablement and after discharge which support improvements in mental health *and* the ability to live as independently as possible. First, existing evidence suggests mental health can impact an individual's capacity to engage in activities which support mental well‐being (e.g. social or other meaningful activities). Second, it can affect capacity, or motivation, to problem solve and manage the activities of daily living (Benbow & Bhattacharyya, 2016; Coll‐Planas et al., 2017; Hjelle, Tuntland, Forland, & Alvsvag, 2017; Lee, 2006; Mlinac & Feng, 2016; Storeng, Sund, & Krokstad, 2018). Given that older age increases the risk of poor mental health, and the associations between mental health and other core outcomes, work to further understand the extent, and how, reablement affects mental health outcomes appears highly pertinent.

### 4.2 Implications of study findings for future economic evaluations

We found the largest contributors to resource use were use of healthcare and social care services and intensity of informal care support. However, most previous studies have looked only at service use. In terms of collecting data on resource use directly from study participants, including informal care support, the SCPQ performed well in terms of completeness of data. However, it is important to note that, where data was collected via home visits, participants typically chose it to be administered as a structured interview rather than self‐complete. Further work is therefore required to assess its suitability if data collection is to be via postal administration.

### 4.3 Including self‐report measures in reablement evaluation

It is now accepted that, where possible, any evaluation of an intervention should include user‐reported outcomes. A key challenge for evaluations of reablement is that recruitment and baseline data collection occurs at a time of frailty or feelings of vulnerability; an issue not uncommon in health and care services research (Gibbons, Black, Fallowfield, Newhouse, & Fitzpatrick, 2016). Incorporating outcomes data collection (both practitioner‐ and self‐reported) into routine practice may offer a partial solution to minimising demands on study participants by avoiding additional data collection visits. However, our and other studies’ findings point to the importance of capturing a range of outcome domains. This may be beyond what services are able to take on in terms of the additional time this requires. Our experiences of using local study staff to collect self‐reported outcomes data are relevant here. Data collection at discharge and at 6 months follow‐up was conducted via a home visit by the same researcher who consented and collected baseline data. This strategy worked well with a very high retention at T_1_. Significant differences in retention at 6 months follow‐up (91% vs. 52%) according to whether home visits or postal administration was used further supports the value of this approach.

### 4.4 Study limitations

Lower than expected recruitment meant a core study objective – comparing models of service delivery – was not fulfilled. The observational study design limits conclusions regarding the observed impacts of reablement on outcomes. However, descriptive data on outcomes – including two outcomes not previously used to evaluate reablement – and resource use, and our experiences of collecting self‐report data, are important and valuable to discuss and share with the research and practice community.

## 5 CONCLUSIONS

Descriptive analysis of outcomes data collected from a cohort of individuals living in three localities in England and receiving reablement from their local reablement service aligns with existing evidence of the positive impacts of reablement. It also suggests that to fully evaluate reablement and understand the mechanisms of change, a range of outcome domains should be assessed over an extended time period. Findings indicate the value of assessing mental health outcomes in future evaluations. Self‐reported outcomes should be a core element of any evaluation (Gibbons et al., 2016) and these were the predominant source of data for this study. Findings regarding patterns of change in outcomes align with other studies, including those using practitioner‐reported measures. Some concerns are raised about the suitability of some existing measures of functioning, and the interpretation of observed changes in social care–related quality of life. As well as collecting data on hospital and social care service use, economic evaluations also need to capture informal care time.
